# Contactin-2, a synaptic and axonal protein, is reduced in cerebrospinal fluid and brain tissue in Alzheimer’s disease

**DOI:** 10.1186/s13195-018-0383-x

**Published:** 2018-06-01

**Authors:** Madhurima Chatterjee, Marta Del Campo, Tjado H. J. Morrema, Matthijs de Waal, Wiesje M. van der Flier, Jeroen J. M. Hoozemans, Charlotte E. Teunissen

**Affiliations:** 1 Neurochemistry Laboratory, Clinical Chemistry Department, VU University Medical Center, De Boelelaan 1117, 1081 HV Amsterdam, the Netherlands; 2 Department of Pathology, VU University Medical Center, Amsterdam, the Netherlands; 3 Alzheimer Center, VU University Medical Center, Amsterdam, the Netherlands; 4 Department of Epidemiology & Biostatistics, VU University Medical Center, Amsterdam, the Netherlands

**Keywords:** Contactin-2, Alzheimer’s disease (AD), CSF biomarker, Tau, Neurogranin, Beta secretase 1

## Abstract

**Background:**

Synaptic and axonal loss are two major mechanisms underlying Alzheimer’s disease (AD) pathogenesis, and biomarkers reflecting changes in these cellular processes are needed for early diagnosis and monitoring the progression of AD. Contactin-2 is a synaptic and axonal membrane protein that interacts with proteins involved in the pathology of AD such as amyloid precursor protein (APP) and beta-secretase 1 (BACE1). We hypothesized that AD might be characterized by changes in contactin-2 levels in the cerebrospinal fluid (CSF) and brain tissue. Therefore, we aimed to investigate the levels of contactin-2 in the CSF and evaluate its relationship with disease pathology.

**Methods:**

Contactin-2 was measured in CSF from two cohorts (selected from the Amsterdam Dementia Cohort), comprising samples from controls (cohort 1, *n* = 28; cohort 2, *n* = 20) and AD (cohort 1, *n* = 36; cohort 2, *n* = 70) using an analytically validated commercial enzyme-linked immunosorbent assay (ELISA). The relationship of contactin-2 with cognitive decline (Mini-Mental State Examination (MMSE)) and other CSF biomarkers reflecting AD pathology were analyzed. We further characterized the expression of contactin-2 in postmortem AD human brain (*n* = 14) versus nondemented controls (*n* = 9).

**Results:**

CSF contactin-2 was approximately 1.3-fold reduced in AD patients compared with controls (*p* < 0.0001). Overall, contactin-2 levels correlated with MMSE scores (*r* = 0.35, *p* = 0.004). We observed that CSF contactin-2 correlated with the levels of phosphorylated tau within the control (*r* = 0.46, *p* < 0.05) and AD groups (*r* = 0.31, *p* < 0.05). Contactin-2 also correlated strongly with another synaptic biomarker, neurogranin (control: *r* = 0.62, *p* < 0.05; AD: *r* = 0.60, *p* < 0.01), and BACE1, a contactin-2 processing enzyme (control: *r* = 0.64, *p* < 0.01; AD: *r* = 0.46, *p* < 0.05). Results were further validated in a second cohort (*p* < 0.01). Immunohistochemical analysis revealed that contactin-2 is expressed in the extracellular matrix. Lower levels of contactin-2 were specifically found in and around amyloid plaques in AD hippocampus and temporal cortex.

**Conclusions:**

Taken together, these data reveal that the contactin-2 changes observed in tissues are reflected in CSF, suggesting that decreased contactin-2 CSF levels might be a biomarker reflecting synaptic or axonal loss.

**Electronic supplementary material:**

The online version of this article (10.1186/s13195-018-0383-x) contains supplementary material, which is available to authorized users.

## Background

Alzheimer’s disease (AD) is the major cause of dementia worldwide [1]. AD patients are characterized by high levels of cerebrospinal fluid (CSF) tau reflecting tangle pathology whereas the underlying amyloid beta (Aβ) plaque pathology is mirrored by decreased levels of Aβ42 in the CSF [2]. However, about 30% of the cognitively normal elderly also have an AD CSF biomarker profile, making AD diagnosis complex [3, 4]. Thus, additional biomarkers are needed for a better diagnosis. Furthermore, synaptic dysfunction [5, 6] and axonal loss [7] are early events in the pathogenesis of AD [6, 8–12]. Synapse loss has been suggested to be related more strongly with cognitive impairment than plaque or tangle pathology [13–16]. Therefore, biomarkers reflecting these changes might be useful to support early diagnosis and prognosis of AD. Several synaptic biomarkers in CSF have been identified, such as neurogranin [17, 18], synaptotagmin [19], synaptosomal-associated protein (SNAP)-25 [20], and Ras-related protein (Rab)-3A [17]. Neurogranin is a promising synaptic biomarker which has been found to be specifically increased in AD [17, 18, 21]. So far, there are no established biomarkers for axonal loss specific for AD. Increased tau level has been related with axonal loss [7], but increased tau is a rather unspecific finding indicating neurodegeneration [22].

Contactin-2 is a soluble cell-adhesion protein primarily expressed on the axonal and synaptic membranes [23–29]. It belongs to the immunoglobulin superfamily and consists of six members (contactin-1 to contactin-6) [29, 30]. Contactin-2 is expressed in hippocampal pyramidal cells, cerebellar granule cells, the juxtaparanodal regions of myelinated nerve fibers [24, 30], and frontal and temporal lobes [23, 31]. Contactin-2 is a multifunctional protein that plays important roles in axonal guidance during development [32, 33], neuronal fasciculation [34], axonal domain organization [35], and neuron-glia interaction [36]. Interestingly, a genome-wide association study (GWAS) identified single nucleotide polymorphisms (SNPs) in the gene encoding contactin-2 (*CNTN2)* associated with AD [36]. Contactin-2 interacts with proteins involved in AD pathogenesis, such as amyloid precursor protein (APP) [37, 38] and beta-secretase 1 (BACE1) [37, 39, 40]. Lower levels of contactin-2 correlated with higher BACE1 activity in postmortem AD tissue [31]. Thus, the interactions between contactin-2 and BACE1 and APP proteins may influence the production of Aβ peptide and the subsequent formation of amyloid plaques. Interestingly, higher levels of contactin-2 have been reported in AD CSF pools using proteomics approaches [37].

We hypothesized that AD might be associated with changes in contactin-2 levels in both CSF and brain. In this study, we aimed to evaluate the potential for contactin-2 as a CSF biomarker candidate reflecting synaptic and axonal dysfunction in AD and to examine its relationship with other important players in AD pathogenesis. Moreover, we further characterized the expression of this protein in postmortem hippocampus to explore the potential role of this protein in AD pathogenesis.

## Methods

### Human CSF sample subjects

For the first study, we included cognitively normal controls with subjective memory complaints (*n* = 28) and AD patients (*n* = 36) (Table 1) from the Amsterdam Dementia Cohort [38]. An additional validation cohort was also included to replicate the findings (controls, *n* = 20; AD, *n* = 70) (Table 1) from the same dementia cohort.

**Table 1:** Demographic details of cohort 1 and cohort 2

	Cohort 1		Cohort 2	
	Controls	Patients with AD	Controls	Patients with AD
*n*	28	36	20	70
Gender (male:female)	13:15	15:21	14:6	29:41
Age (years) (mean ± SD)	60 ± 7	62 ± 6	62 ± 3	62 ± 5
MMSE (mean ± SD)	27 ± 3	19 ± 5***	28 ± 2	20 ± 6***
Aβ42 (pg/ml) (median [IQR])	915 [815–1026]	468 [395–552]***	1063 [1009–1214]	578 [518–645]***
tTau (pg/ml) (median [IQR])	216 [161–309]	691 [559–962]***	274 [239–315]	734 [552–1021]***
pTau (pg/ml) (median [IQR])	47 [33–54]	92 [77–116]***	43 [39–50]	90 [69–107]***
Contactin-2 (ng/ml) (median [IQR])	78 [69–110]	59 [42–74]***	65 [54–99]	61 [39–78]*

*Aβ* amyloid beta, *AD* Alzheimer’s disease, *IQR* interquartile range, *MMSE* Mini-Mental State Examination, *SD* standard deviation

*p < 0.05, **p < 0.01, ***p < 0.001, versus controls

Diagnoses were defined in a multidisciplinary committee according to the criteria of the National Institute of Neurological and Communicative Disorders and Stroke–Alzheimer’s Disease and Related Disorders Association [39, 40]. AD cases were additionally selected based on a positive AD biomarker profile (CSF total tau (tTau)/Aβ42 ratio > 0.52 [41]). When patients presented with cognitive complaints and the results of clinical assessments were within the normal range they were labeled as subjective memory complaint, hereinafter referred to as controls [18]. These nondemented control cases were selected based on a negative CSF AD biomarker profile (CSF tTau/Aβ42 ratio < 0.52 [41]). CSF was collected by standard lumbar puncture and stored according to previously published JPND-BIOMARKAPD guidelines until analysis [18, 42]. Samples within each cohort were matched for age. Demographic and clinical details of all patients are listed in Table 1.

### Postmortem brain tissue

Postmortem hippocampus and temporal cortex from AD patients (*n* = 14) and nondemented controls (*n* = 9) were obtained from the Netherlands Brain Bank. Considering that contactin-2 is expressed in the hippocampus [30, 43] and temporal cortex [23, 31], and these brain areas are primarily affected in AD [44], we used homogenates of postmortem hippocampus and temporal cortex tissue. All donors or their next of kin provided written informed consent for brain autopsy and the use of medical records for research purposes. Sample processing is described in detail in Additional file 1: Section 1.1. Patient details such as clinical and pathological diagnosis, Braak stage, age and sex, and postmortem delay are outlined in Additional file 1: Table S1.

### Enzyme-linked immunosorbent assay (ELISA) analysis

Contactin-2 was measured in both CSF and postmortem brain tissue homogenates with the Contactin-2 duoset ELISA kit (R&D, Minneapolis, USA; cat. nos. DY1714–05 and DY008), which uses antibodies raised against the secreted part of contactin-2 (Leu29-Asn1012). We validated this kit both for CSF samples and tissue samples using previous validation guidelines [45, 46] (Additional file 1: Table S2). CSF and postmortem brain samples were diluted 1:16 and 1:100, respectively, in reagent diluents provided in the kit and the assay was performed according to the manufacturer’s protocol. The intra-assay percentage coefficients of variation (%CVs) for CSF and brain tissue were 1.9 and 1.3, and the interassay %CVs were 8.7 (CSF) and 9 (brain tissue), respectively. Specifics about the procedure can be found in Additional file 1: Section 1.2. CSF Aβ42, tTau, and phosphorylated tau (pTau) were measured as a part of routine diagnosis at the Neurochemistry laboratory at VU University Medical Centre, Amsterdam, the Netherlands, using commercially available ELISA (Fujirebio, Ghent, Belgium) as previously performed [47] (Table 1). CSF BACE1 and neurogranin were measured using commercially available analytically validated ELISA kits from Euroimmun (Lübeck, Germany). CSF Aβ40 was measured using the V-PLEX Plus Aβ Peptide Panel 1 (6E10) Kit (MSD, Maryland, USA). All samples were randomized and were measured by a single experienced technician blinded to the clinical groups.

### Western blotting

Human hippocampus and temporal cortex tissue homogenates (20 μg per sample) were prepared in sample buffer (2% SDS, 0.03 M Tris, 5% 2-mercaptoethanol, 10% glycerol, bromophenol blue) and heated for 5 min at 95 °C. Electrophoresis was carried out using 10% SDS-PAGE minigels. Next, proteins were transferred to polyvinylidene fluoride (PVDF) membranes (Millipore, Bedford, USA) that were subsequently blocked for 30 min with blocking buffer (5% w/v nonfat dried milk in PBS-Tween 0.5% v/v (PBS-T)), and incubated with the corresponding primary antibodies—affinity-purified polyclonal rabbit anti-Contactin-2 [48] (1:1500, SAB4200299; Sigma Aldrich, St. Louis, USA) or monoclonal rabbit anti-GAPDH (1:1000, clone 14C10; Cell Signaling Technology, MA, USA)—overnight at 4 °C. After washing with wash buffer (0.05% w/v milk in TBS-T), membranes were incubated for 1 h with polyclonal goat anti-rabbit IgG/HRP (1:2000, DAKO, Glostrup, Denmark) or goat anti-mouse IgG/HRP (1:1000, DAKO) in blocking buffer. Protein bands were detected with the ECL Western Blotting detection kit (GE Healthcare, Amersham, UK). Samples were always randomly distributed within the gels and the researcher was unaware of the diagnosis and specifics of the samples. Immunoblot films were scanned, and signal quantification was performed using ImageJ 1.45 (NIH, Bethesda, USA). Contactin-2 band signal was normalized by the GAPDH signal intensity.

### Immunohistochemistry and immunofluorescence

Formalin-fixed and paraffin-embedded hippocampus and temporal cortex sections (5 μm) were mounted on Superfrost plus tissue slides (Menzel-Glaser, Braunschweig, Germany) and dried overnight at 37 °C. Samples from 12 individuals (7 AD and 6 controls) were immunostained. Two sections from each subject were analyzed  and stainings were found to be consistent. Immunohistochemistry (IHC) and immunofluorescence (IF) procedures are described in detail in Additional file 1: Section 1.3. The primary antibodies used were: affinity-purified polyclonal rabbit anti-Contactin-2 (IHC: 1:400, IF: 1:25; HPA001397, Atlas Antibodies, Stockholm, Sweden); monoclonal mouse anti- pTau Ser202/Thr205 AT-8 (IF: 1:800, MN1020, Thermo Fisher Scientific, Landsmeer, Netherlands); and monoclonal mouse anti-Aβ IC-16 (IF: 1:200, a kind gift from Dr. Korth, University of Duesseldorf, Germany). For IHC, the bound primary antibody was detected using DAKO anti-rabbit/mouse EnVision+ System-HRP (DAKO, 45007, Glostrup, Denmark). Nuclei were visualized by Mayer’s hematoxylin counterstain (Merck, MHS1, Zwijndrecht, Netherlands). For IF, the following secondary antibodies were used: anti-rabbit alexa-647 (1:250, DAKO), anti-mouse alexa-488 (1:250, DAKO), and anti-mouse alexa-594 (1:250, DAKO). Thioflavin-S (Merck, T1892) was added to brain tissue sections after incubation with primary anti-pTau and anti-contactin-2 antibodies and corresponding secondary antibodies for 1 min with a prior 10-min acetone fixation at room temperature. The slides were finally incubated with DAPI for 10 mins and subsequently covered using 80% glycerol in TBS, pH 7.4. Staining and imaging was performed by two independent researchers who were unaware of the diagnosis of the cases. IHC images were captured with a Zeiss light microscope equipped with a digital camera and a 10× or 25× objective (12.5× ocular). IF images were captured with a Nikon Eclipse Ti confocal microscope equipped with a 60× oil (numerical aperture (NA) = 1.40) objective and NisElements 4.30 software.

### Statistics

Differences in CSF contactin-2 levels between groups were tested with analysis of covariance (ANCOVA) adjusted for age and gender when applicable. Data were normalized by Templeton’s two-step method [49] if not normally distributed. Correlation analyses were performed using Pearson or Spearman correlation for parametric and nonparametric data, respectively. Group differences between AD and controls in postmortem samples were evaluated by Mann-Whitney *U* test.

The statistical tests were two-tailed and values with *p(two-tailed)* < 0.05 were considered significant. Statistical analyses were performed on SPSS version 22 (IBM SPSS Statistics for Windows, Version 21.0; IBM Corp., Armonk, NY, USA). Graphs were plotted using GraphPad Prism version 6.07.

## Results

### Contactin-2 CSF levels decreased in AD patients

**Fig. 1:**
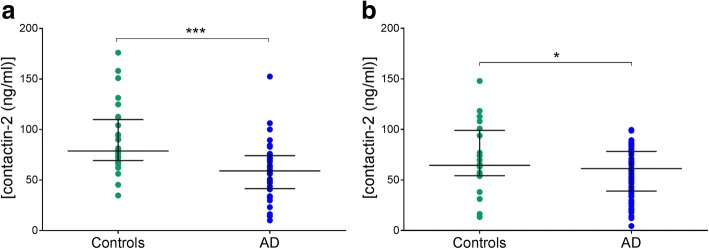
Contactin-2 levels in the CSF. **a**. Scatterplot showing CSF contactin-2 levels in nondemented controls with subjective memory complaints (controls, *n* = 28) and patients with Alzheimer’s disease (AD, *n* = 36). **b**. Contactin-2 levels in controls (*n* = 20) and AD patients (*n* = 70) in a second validation cohort. The values are presented as medians with interquartile ranges. Data were adjusted for age and gender. *p < 0.05, ***p < 0.0001

Demographic and biomarker characteristics of all cases are listed in Table 1. CSF contactin-2 was reduced by 38% in AD patients compared with controls (*p* < 0.0001; Fig. 1a). This result was further validated in a second cohort (*p* = 0.049; Fig. 1b), where contactin-2 was reduced by 20%. A positive correlation was observed between CSF contactin-2 and the Mini-Mental State Examination (MMSE) in the total group (*r* = 0.35, *p* = 0.004; Additional file 1: Figure S1). The correlation between contactin-2 and MMSE were not significant when AD and control groups were analyzed separately. The correlation was not observed in the second cohort (*r* = 0.11, *p* = 0.2).

### CSF contactin-2 and its relationship with core AD biomarkers

**Fig. 2:**
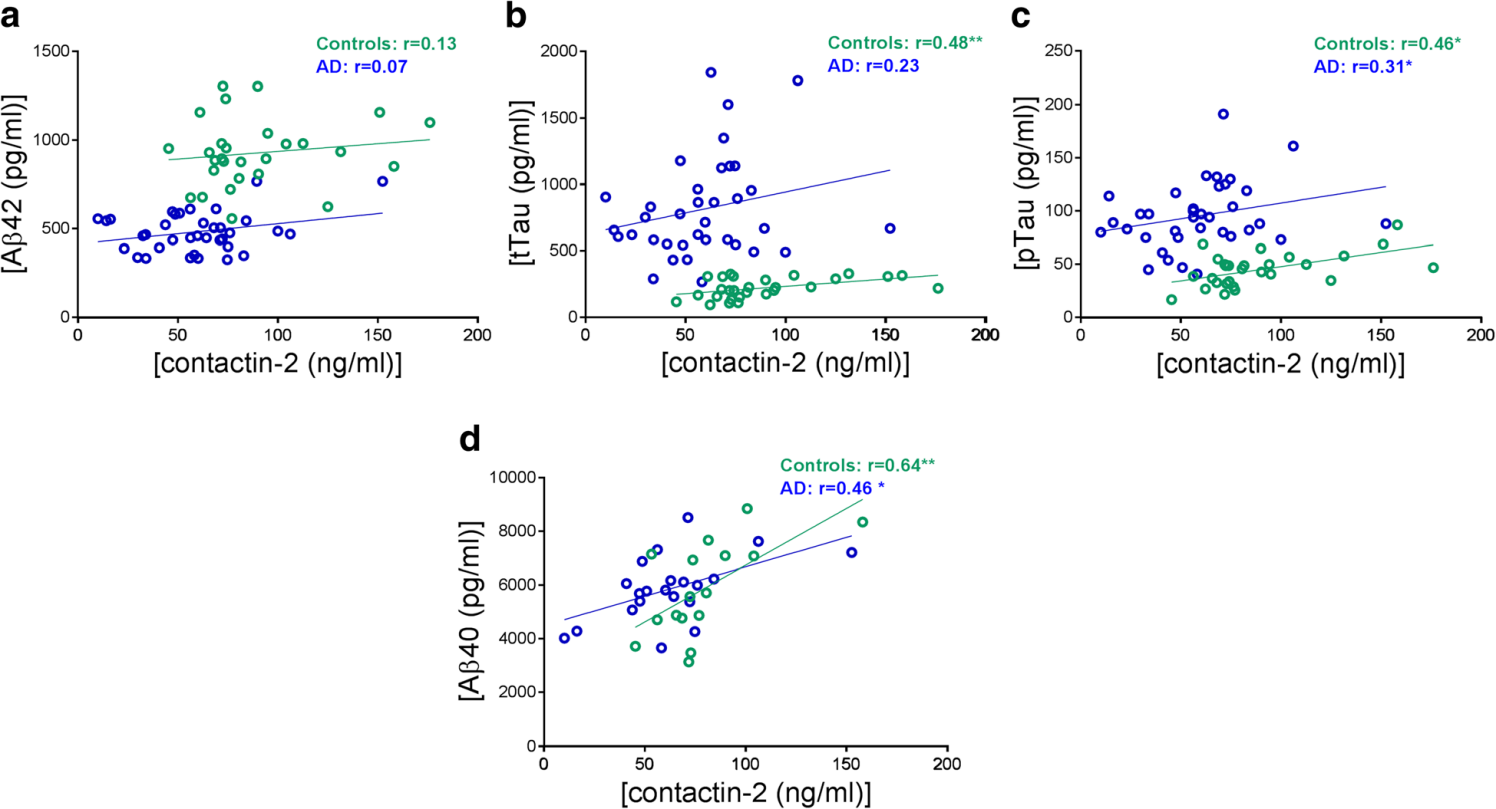
Correlations of CSF contactin-2 levels with **a.** amyloid beta (Aβ)42, **b.** total tau (tTau), **c.** phosphorylated tau (pTau), and **d.** Aβ40. *p < 0.05, **p < 0.01. AD Alzheimer’s disease

Correlations were analyzed within each diagnostic group (controls and AD individually). No correlation between contactin-2 and Aβ42 was observed within the control or AD groups (Fig. 2a). Both tTau and pTau strongly correlated with contactin-2 within controls (tTau: *r* = 0.48, *p* = 0.009, Fig. 2b; pTau: *r* = 0.46, *p* = 0.01, Fig. 2c). Within the AD groups only pTau correlated positively with contactin-2 (*r* = 0.31, *p* = 0.05, Fig. 2c). Similar results were obtained in the second cohort, with the exception of tTau which now correlated with contactin-2 within both control and AD groups (Additional file 1: Figure S2). Additionally, contactin-2 also correlated with CSF Aβ40 within both groups (*n* = 37; controls: *r* = 0.64, *p* = 0.008; AD: *r* = 0.46, *p* = 0.03, Fig. 2d). Since contactin-2 was associated with age within the AD group in the second cohort, an age correction was applied (Additional file 1: Figure S2).

### Contactin-2 correlates with neurogranin and BACE-1

**Fig. 3:**
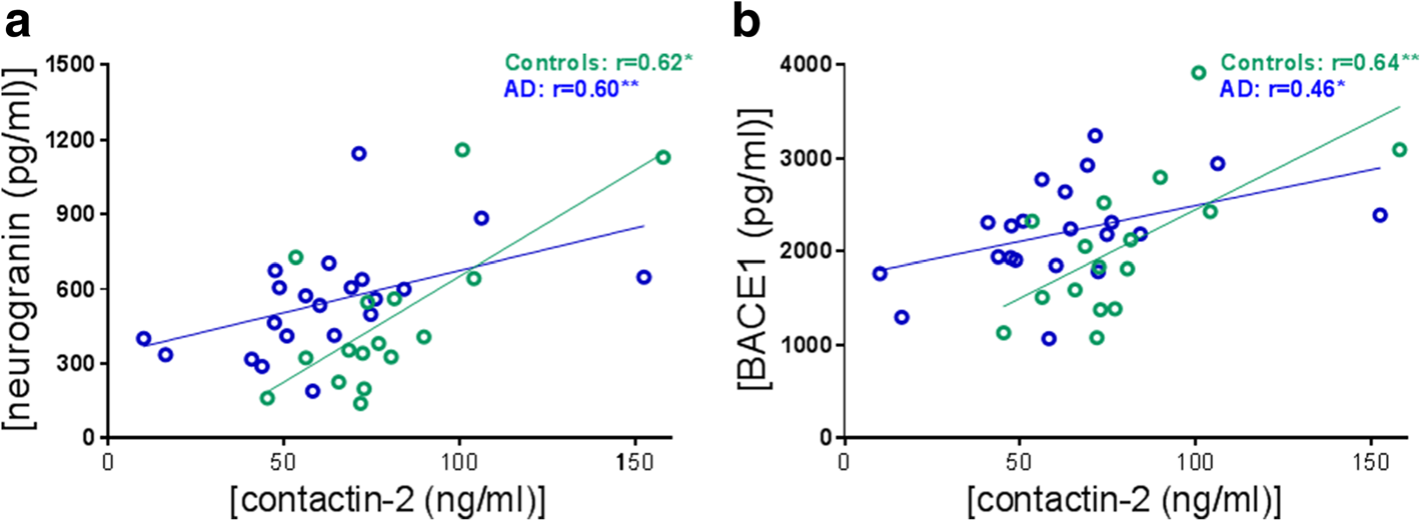
Correlations of CSF contactin-2 with **a.** CSF neurogranin and **b.** CSF beta-secretase 1 (BACE1). *p < 0.05, **p < 0.01. AD Alzheimer’s disease

To explore the role of contactin-2 in synapse loss, we investigated the relationship of contactin-2 with an established synaptic biomarker, neurogranin. Contactin-2 correlated strongly with CSF neurogranin within controls and AD (controls: *r* = 0.62, *p* = 0.01; AD: *r* = 0.60, *p* = 0.004, Fig. 3a). Furthermore, we analyzed the correlation of contactin-2 with its processing enzyme BACE1. Strong correlations between contactin-2 and CSF BACE1 were present within controls and AD (controls: r = 0.64, *p* = 0.007; AD: r = 0.46, *p* = 0.04, Fig. 3b). These results were validated in the second cohort (Additional file 1: Figure S3). Since contactin-2 and BACE1 both were associated with age within the AD group in the second cohort, an age correction was applied in the corresponding correlation analyses (Additional file 1: Figure S3).

### Characterization of contactin-2 in postmortem human hippocampus and temporal cortex

**Fig. 4:**
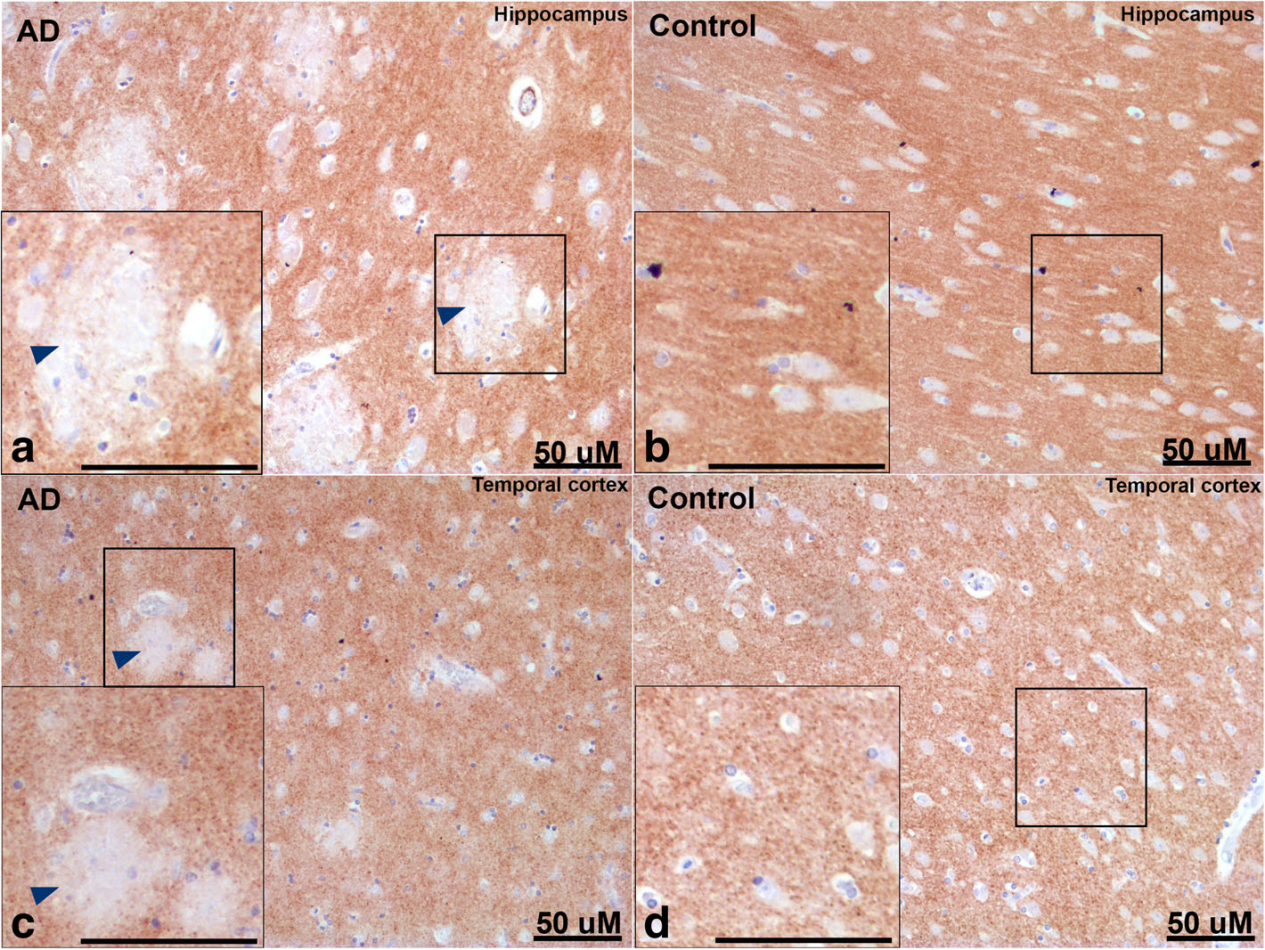
Immunohistochemistry on postmortem human brain sections. Brain sections (**a,b** hippocampus; **c,d** temporal cortex) of subjects with Alzheimer’s disease (AD) (**a,c**) and control subjects (**b,d**) were stained with anti-contactin-2 antibody. Areas with reduced contactin-2 staining are clearly visible in AD brain sections possibly in and around areas with amyloid plaques (shown by arrowheads)

**Fig. 5:**
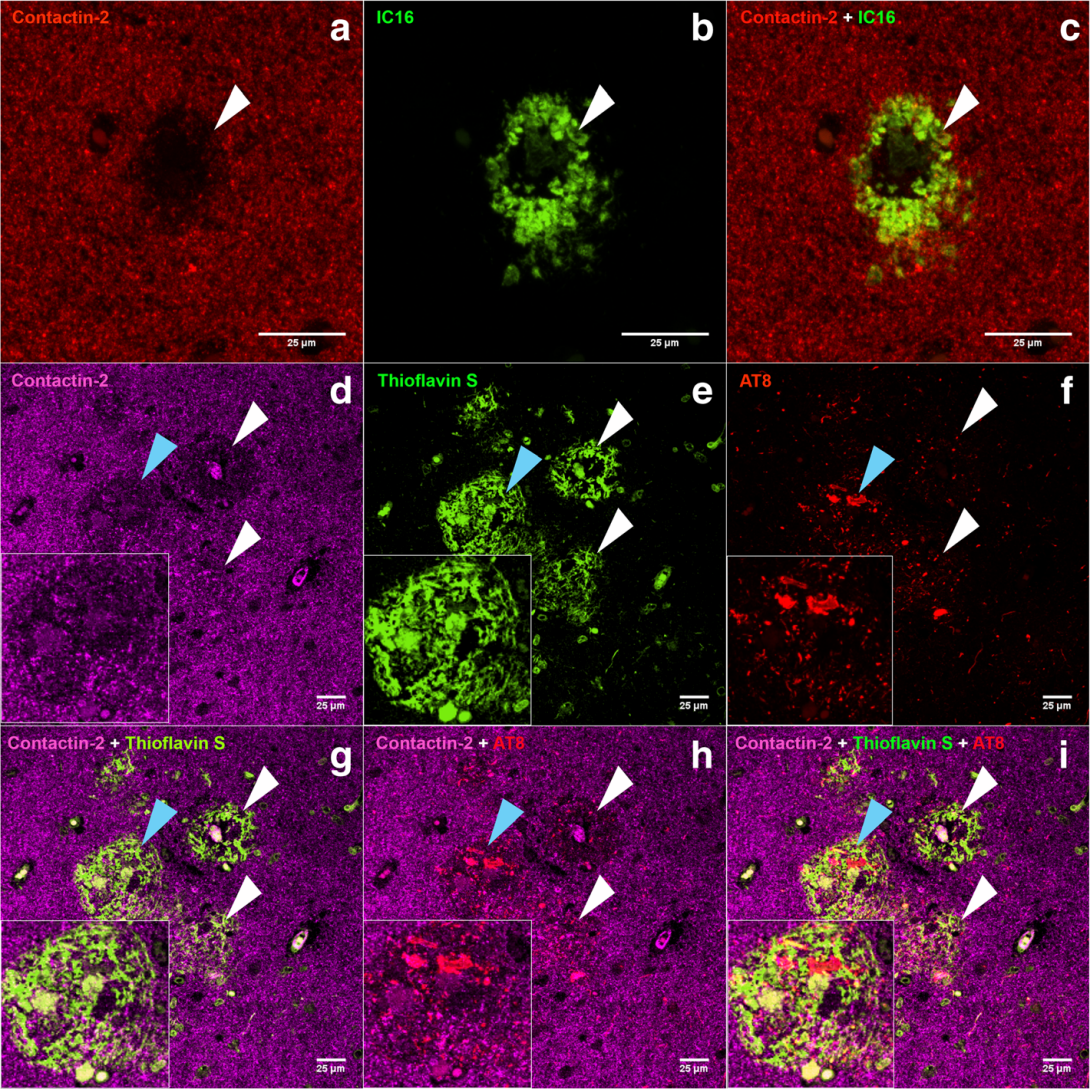
Immunolabeling of contactin-2, Aβ42, and pTau in hippocampal postmortem human AD brain sections. CA subiculum areas of hippocampal sections stained with anti-contactin-2 (**a**,**d**), anti-Aβ42 (**b**), thioflavin S (**e**), and anti-phosphorylated tau (**f**). Merged images are shown in **c.** (contactin-2 + IC16), **g.** (contactin-2 + thioflavin S), **h.** (contactin-2 + AT8), and **i.** (contactin-2 + thioflavin S + AT8). Areas with reduced contactin-2 expression (shown by white arrows) can be seen in AD brain sections in and around areas with neuritic amyloid plaques. Areas marked with blue arrows have been magnified in the inserts

Immunohistochemistry showed that contactin-2 was mainly expressed in the extracellular matrix in both control and AD groups in postmortem hippocampus and temporal cortex (Fig. 4). Interestingly, within the AD cases we observed a specific reduction in contactin-2 staining in areas resembling amyloid plaques (Fig. 4a, c, where probable plaques are shown by arrowheads). We next analyzed the potential relationship of contactin-2 expression with the main hallmarks of AD (Fig. 5). Areas with reduced contactin-2 staining contained deposits of Aβ (Fig. 5a–c), as well as pTau- and Thioflavin S-positive structures (Fig. 5d–i), which indicates the reduction of contactin-2 staining in fibrillar neuritic plaques.

**Fig. 6:**
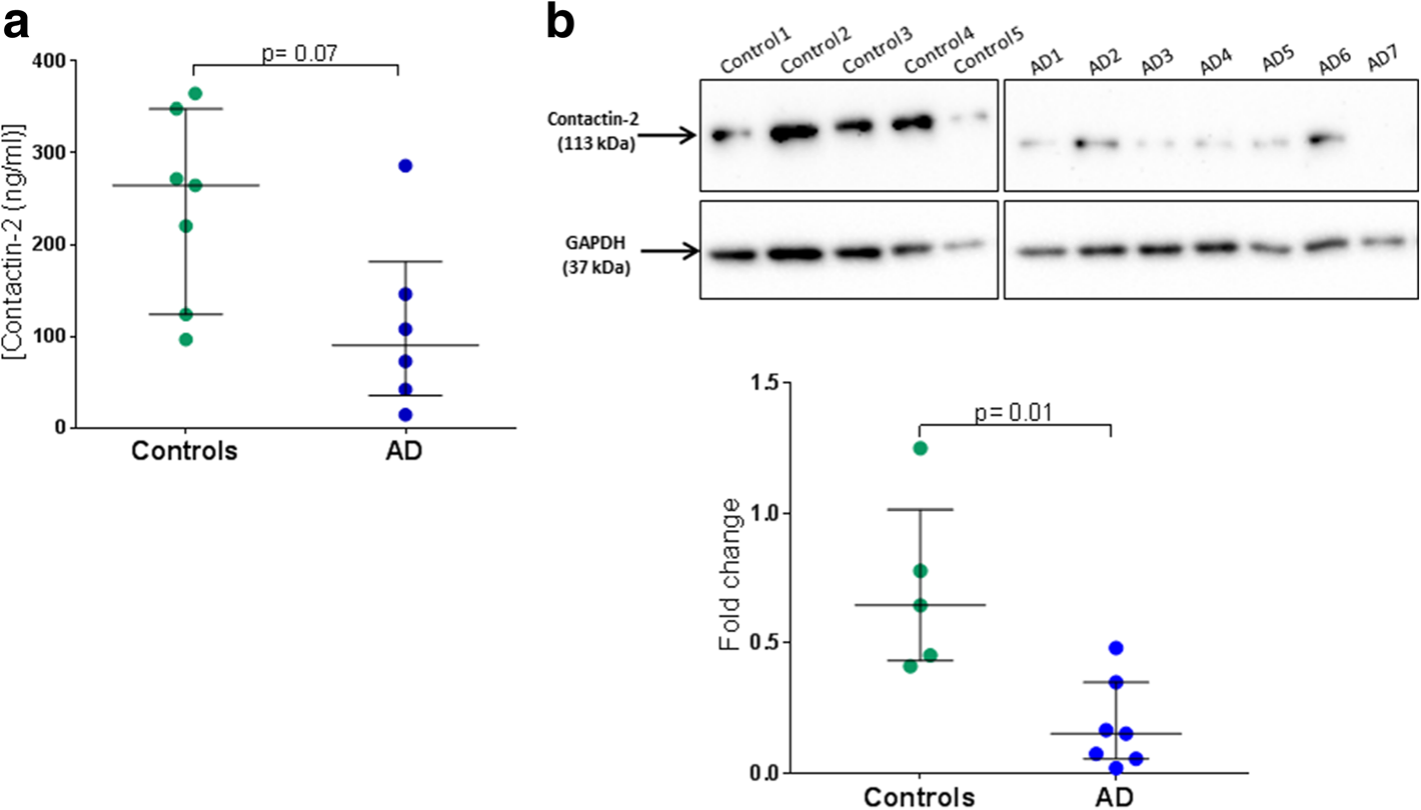
Contactin-2 levels in postmortem hippocampus of Alzheimer’s disease (AD) patients versus controls. **a.** Contactin-2 concentration measured by ELISA and corrected for total protein concentration. The values are presented as medians with interquartile ranges. **b.** Western blot showing contactin-2 levels normalized with GAPDH in AD versus controls. Full image of the Western blot is shown in Additional file 1: Figure S5. Unpaired *t* test was used for group comparisons

Analysis of postmortem tissue homogenates by ELISA confirmed that contactin-2 tended to be decreased in AD hippocampus (*n* = 7) compared with controls (*n* = 6, *p* = 0.07) (Fig. 6a). However, Western blot analysis revealed a significant reduction in contactin-2 levels since the expected 113-kDa contactin-2 band decreased in AD (*n* = 7) compared with controls (*n* = 5, *p* = 0.01; Fig. 6b and Additional file 1: Figure S5).

## Discussion

The main finding of this study is that the levels of the synaptic/axonal protein contactin-2 in the CSF differs between AD patients and controls, and is associated with other biomarkers, particularly tTau, pTau, Aβ40, BACE1, and neurogranin. Moreover, we also performed characterization of this protein in postmortem human brain tissue and found areas with reduced contactin-2 expression in and around fibrillar neuritic plaques.

Synaptic dysfunction and axonal loss are early events in AD preceding cognitive decline [5, 7]. Detection of changes related to these mechanisms may therefore contribute to early diagnosis of the disease. Our findings in the CSF reveal that contactin-2 is reduced in AD cases compared with controls in two cohorts, which challenges previous proteomics findings that identified increased levels of this synaptic protein in three pooled AD CSF samples [37]. However, the use of specific antibody-based technologies detecting very specific epitopes of contactin-2 in the current study may explain the observed discrepancies. Even though CSF contactin-2 levels were lower in AD patients compared with controls, there was a substantial overlap between the groups in both cohorts which may limit its diagnostic performance. Contactin-2 levels may even be increased in the early stages of AD and then decrease with disease severity as has been shown in longitudinal analysis of other neuronal injury markers [50]. Considering that synaptic/axonal changes occur in very early stages of the disease, it would be of interest to explore whether stronger or opposite changes are observed at earlier stages of the disease, and to study its potential as a diagnostic and prognostic marker for early AD. Interestingly, similar to the changes in CSF, contactin-2 levels were decreased in postmortem brain tissue of AD cases compared with controls. Our results are supported by a previous study that found a reduction in contactin-2 in hippocampal brain tissue homogenates of selected AD patients with high BACE1 activity compared with age-matched controls [31]. Therefore, these results not only indicate that contactin-2 is changed in the AD brain but also that such changes are reflected within the CSF, highlighting the potential of this protein as a novel biomarker for loss of synaptic/axonal integrity.

Synaptic biomarkers such as neurogranin have been suggested to reflect cognitive decline [18, 51]. In this study, we observed a correlation of CSF contactin-2 with MMSE, suggesting a possible relationship between contactin-2 and cognition. However, this could not be validated in the second cohort. Nonetheless, we found a strong correlation between contactin-2 and neurogranin, supporting the role of contactin-2 in synaptic dysfunction.

CSF contactin-2 correlated with tTau and pTau within the AD/control groups, being stronger within the control group, which suggests that contactin-2 is a sensitive marker reflecting general axonal loss and changes in tau homeostasis under normal physiological conditions. Immunohistochemical characterization of contactin-2 performed in postmortem brain tissue showed a reduction in contactin-2 expression in areas with neuritic amyloid plaques, characterized by thioflavin S, pTau, and Aβ staining. Therefore, similar to the findings in CSF, contactin-2 expression was also found to be related with tau in brain tissue, supporting the potential role of contactin-2 in axonal loss and incipient neurodegeneration.

**Fig. 7:**
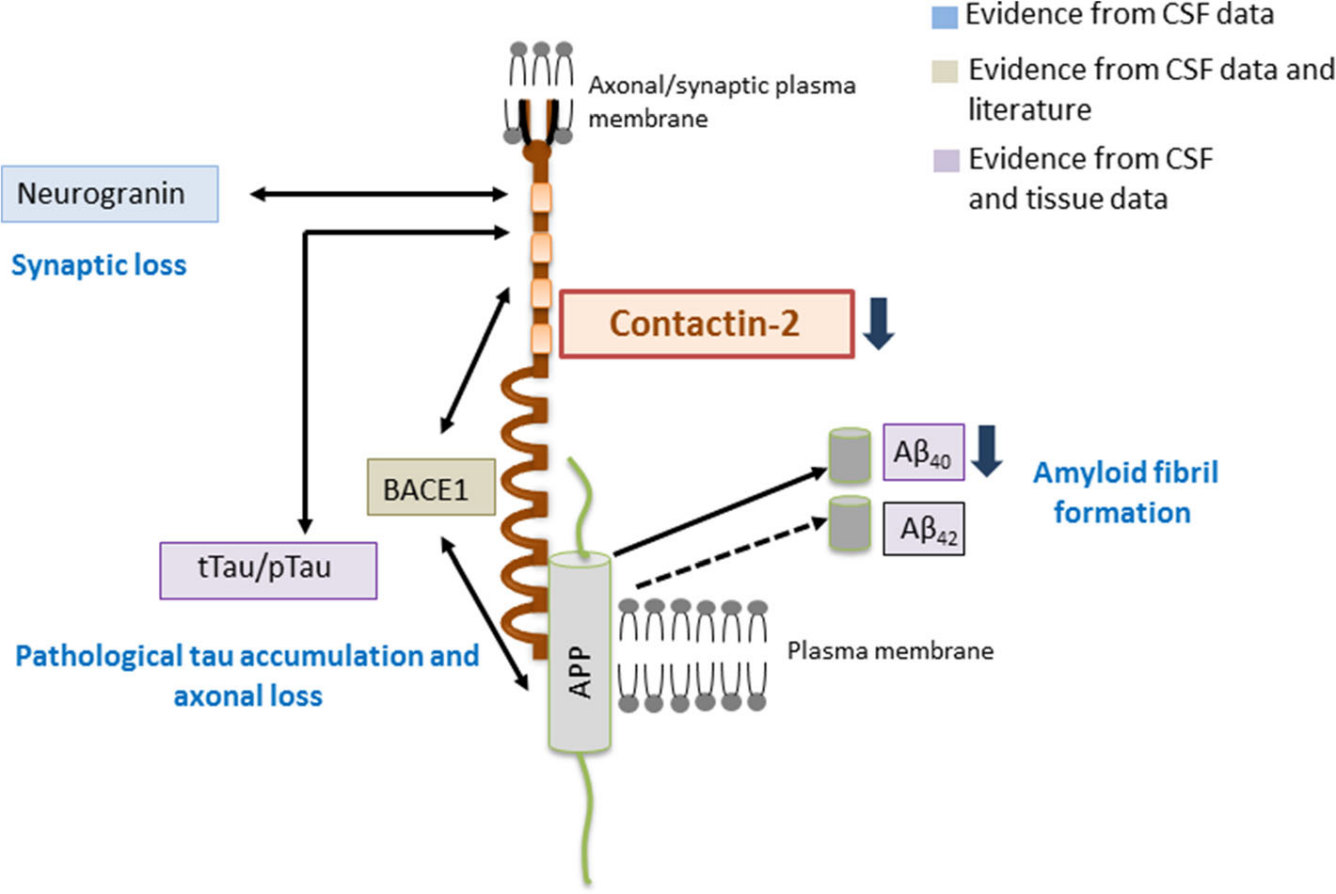
Schematic summary of the hypothesis. Contactin-2 interacts with beta-secretase 1 (BACE1) and amyloid precursor protein (APP). Binding of contactin-2 with APP leads to APP processing and amyloid beta (Aβ) peptide release. Based on our data, we hypothesize that a decrease in contactin-2 levels (shown by a thick dark blue arrow) in AD might be a cellular protective mechanism to reduce the binding of contactin-2 with APP and thus subsequently lowering production of Aβ. Correlations of contactin-2 with total tau (tTau)/phosphorylated tau (pTau) and neurogranin suggest possible interactions among these molecules or their involvement in common pathogenic mechanisms. Solid single/double headed arrows indicate correlations/interactions between two proteins and dashed arrow indicates no correlation. CSF cerebrospinal fluid

CSF contactin-2 strongly correlated with Aβ40 and BACE-1, suggesting an association between contactin-2 and Aβ production. This is supported by previous studies showing that binding of contactin-2 to APP [52, 53] enhances the production of the APP intracellular domain (AICD) in the cytosol with concomitant Aβ peptide generation [54, 55] (Fig. 7). Interestingly, thioflavin S-positive fibrillar plaques that show a stronger presence of Aβ40 than Aβ42 [56] had lower contactin-2, probably as a protective mechanism to avoid Aβ40 formation in those areas. We did not observe a strong absence of contactin-2 in areas with diffuse plaques (data not shown) that primarily consist of Aβ42 [56]. Similarly, correlation with Aβ42 was lacking in the CSF. Taken together, these data suggest that contactin-2 can influence the homeostasis of Aβ which may ultimately affect the formation of amyloid deposits and the pathogenesis of AD. The decrease in contactin-2 levels in AD might be a cellular protective mechanism to reduce the binding of contactin-2 with APP and thus subsequently lowering production of Aβ (Fig. 7).

It should be noted that we observed positive correlations between contactin-2 and tau, BACE1, and neurogranin, which is the opposite to what can be expected on the basis of usually increased CSF levels of the latter proteins in AD. This indicates that contactin-2 is physiologically associated with these proteins strongly, demonstrated by high positive correlations within controls, and that a disease pathology such as AD possibly disrupts these associations making the correlations weaker in the CSF of AD patients. In addition, these discrepancies may occur because the correlations were analyzed within the AD and control groups separately rather than as a whole cohort. In the whole cohort, there is indeed a tendency towards negative correlation with Tau as expected (cohort 1, *r* = − 0.23, *p* = 0.09; Additional file 1: Figure S7). CSF BACE was not significantly changed in AD versus controls. Thus, a pattern in correlation may not be evident.

One limitation of this case-control study was the relatively small sample size, even though we eventually included 106 AD patients and 50 controls in the total group. Controls with subjective memory complaints, AD patients, or patients with other neurodegenerative disorders often present similar clinical symptoms [57] which might obscure the differences between the different clinical groups. However, AD patients were selected by clinicians from a specialized memory center based on the cut-off value for CSF tau to Aβ42 ratio [41] ensuring a more reliable diagnosis based on fluid biomarkers [58]. Another limitation of the study was that no cohort was used from another memory clinic. It would be interesting to investigate the levels of contactin-2 in larger independent cohorts and in AD patients in different stages of the disease.

## Conclusions

In summary, this study reveals a reduction in the axonal and synaptic protein contactin-2 in two CSF cohorts and postmortem tissue, and indicates the potential of this protein as a novel AD CSF biomarker reflecting synaptic/axonal dysfunction. Future studies should investigate how contactin-2 is changed during the course of AD in a longitudinal study design with larger patient cohorts. In addition, studies revealing a mechanistic relation between contactin-2, Aβ, and tau are required to understand the bigger picture of the cell signaling pathway underlying AD pathogenesis and to open new leads for therapy development.

## Additional file:

Additional file 1: Supplementary methods and results. (DOCX 645 kb)

## Abbreviations

AD: Alzheimer’s disease
AICD: Amyloid precursor protein intracellular domain
APP: Amyloid precursor protein
Aβ: Amyloid beta
BACE1: Beta-secretase 1
CSF: Cerebrospinal fluid
CV: Coefficient of variation
ELISA: Enzyme-linked immunosorbent assay
IF: Immunofluorescence
IHC: Immunohistochemistry
MMSE: Mini-Mental State Examination
pTau: Phosphorylated tau
tTau: Total tau
