# Supplementary material for: Contactin-2, a synaptic and axonal protein, is reduced in cerebrospinal fluid and brain tissue in Alzheimer’s disease
Source: Alzheimers Res Ther. 2018 Jun 1;10:52. doi: 10.1186/s13195-018-0383-x (PMC5984818; doi:10.1186/s13195-018-0383-x)
Supplement: Supplementary file 1 — Supplementary methods and results. (DOCX 645 kb) [file 13195_2018_383_MOESM1_ESM.docx]

**Additional file 1**

**1. Materials and Methods**

***1.1. Clinical, neuropathological evaluation and human brain tissue sample processing***

***Post-mortem brain tissue***

Clinical diagnosis was defined according to DSM-III-R criteria and the severity of dementia prior to death had been evaluated with the Global Deterioration Scale of Reisberg(Reisberg et al. 1982). Neuropathological evaluation was performed on formalin-fixed, paraffin-embedded tissue from different brain areas. The distribution and the density of neurofibrillary tangles (NFTs) were determined using Bodian staining and immunohistochemistry for hyperphosphorylated tau. Staging of Alzheimer’s disease was evaluated according to the Braak criteria for NFTs( Braak and Braak 1991; Braak et al. 2006) and according to Thal criteria for amyloid deposition(Thal et al. 2002).

Frozen human brain hippocampus tissue and temporal cortex tissue blocks were present from 13 of these cases. The tissue was homogenized with T-per Extraction Reagent (T-Per, 0.1g/ml, Thermo Scientific, Waltham, USA) containing EDTA-free Protease Inhibitor Cocktail (1:25, Roche, Basel, Switzerland). Tissue homogenates were centrifuged at 10500 g for 30 min at 4°C. The protein content in the supernatant was quantified using bovine serum albumin (BSA) standards (Thermo Scientific, Waltham, USA) and the Bio-Rad Protein Assay (Bio-Rad, Hercules, USA). Samples were stored at -80ºC until further analysis.

**Table S1** List of Clinical and pathological diagnoses of all cases

| **Subjects** | **Tau Braak** | **Aβ Braak** | **Gender (F/M)** | **Age (years)** | **Post-mortem delay (hours)^$^** | **Clinical Diagnosis** | **IHC/WB^#^** |
| --- | --- | --- | --- | --- | --- | --- | --- |
| 1 | 6 | C | F | 62 | 4.45 | AD | IHC |
| 2 | 6 | C | F | 87 | 8.00 | AD | IHC |
| 3 | 6 | C | F | 82 | 5.30 | AD | IHC |
| 4 | 6 | C | M | 74 | 7.40 | AD | IHC |
| 5 | 6 | C | F | 81 | 6.00 | AD | IHC |
| 6 | 5 | C | F | 84 | 4.50 | AD | IHC |
| 7 | 6 | C | M | 73 | 6.15 | AD | IHC |
| 8 | 5 | C | F | 75 | - | AD | WB |
| 9 | 6 | C | F | 76 | - | AD | WB |
| 10 | 6 | C | F | 70 | - | AD | WB |
| 11 | 6 | B | M | 65 | - | AD | WB |
| 12 | 2 | 0 | M | 60 | - | AD | WB |
| 13 | 3 | B | F | 90 | - | AD | WB |
| 14 | 6 | C | F | 69 | - | AD | WB |
| 15 | 1 | 0 | F | 75 | 5.25 | Control | IHC and WB |
| 16 | 2 | B | F | 84 | 6.05 | Control | IHC |
| 17 | 2 | B | F | 83 | 4.40 | Control | IHC and WB |
| 18 | 1 | 0 | M | 82 | 5.50 | Control | IHC |
| 19 | 1 | B | F | 85 | 7.05 | Control | IHC |
| 20 | 1 | 0 | F | 60 | 6.50 | Control | IHC |
| 21 | 2 | B | M | 87 | - | Control | WB |
| 22 | 1 | 0 | M | 57 | - | Control | WB |
| 23 | 4 | B | M | 83 | - | Control | WB |

*^#^Samples used for immunohistochemistry IHC/Western blotting (WB) have been indicated.*

*^$^Exact Post-mortem delay times were unavailable for some samples but the average delay was 6 hours.*

***1.2. Contactin-2 sandwich ELISA***

All reagents were provided in the ELISA kit and they were diluted according to the manufacturer’s protocol. Briefly, a 96-well ELISA plate was coated with the capture antibody (4 ug/mL) and incubated overnight at room temperature (RT). Plate was then washed three times with 1X wash buffer (300 uL for each well). A seven-point standard curve (0.156 ng/mL, 0.313 ng/mL, 0.625 ng/mL, 1.25 ng/ml, 2.5 ng/mL, 5 ng/mL and 10 ng/mL) . CSF samples were diluted 1:16 and hippocampus and temporal cortex tissue homogenates were diluted 1:50 and 1:100 respectively with reagent diluent and incubated at RT for 2 hours on gentle shaking. Following steps were carried out according to the manufacturer’s protocol.

***Table S2*** *Contactin-2 ELISA validation results*

|  | **CSF** | **Brain tissue homogenate** |
| --- | --- | --- |
| LLOQ | 210 pg/ml | 250 pg/ml |
| Intra-assay %CV | 1.9 | 1.3 |
| Inter-assay %CV | 8.7 | 9 |
| Overall % linearity | 87 (acceptable range 80-120%) | 101 (acceptable range 80-120%) |
| Overall in range% | 82 (acceptable range 80-120%) | 99 (acceptable range 80-120%) |
| % Spike recovery | 98 (acceptable range 80-120%) | 99 (acceptable range 80-120%) |

***1.3. Immunohistochemistry (IHC) and immunofluorescence (IF) staining***

For all stainings, sections were deparaffinized with xyleen (3x5 min) and alcohol (4x 2-5 min) and subsequently immersed in 0.3% H2O2 in PBS for 30 minutes to quench endogenous peroxidase activity. The quenching step was skipped if fluorescent antibodies are used. The sections were washed in running tap water. Sections were boiled in an autoclave in 10 mmol/L pH 6.0 sodium citrate buffer. The slides were cooled down and washed in demi-water for 2 minutes and followed by PBS wash for 5 minutes. The slides were incubated with primary antibody diluted in (100 µl per tissue) at RT overnight.

For IF staining, secondary antibodies were added after primary antibody and washing step. The slides were washed in PBS for 5 minutes and then incubated with 100 µl 1:10000 DAPI in PBS for 10 minutes followed by 2X 5 minutes PBS wash. Slides were rinsed with PBS 5 times. The slides were covered with coverslips with TRIS buffered 80% glycerol.

**2. Results**

**Figure S1: Correlations of CSF contactin-2 levels with MMSE in cohort 1.** The regression lines in the figures are for visualization purposes. r=Spearman’s correlation coefficient. ***p* < 0.01

**Figure S2: Correlations of CSF contactin-2 levels with Aβ42, total tau (tTau) and phosphorylated tau (pTau) in validation cohort (cohort 2).** Scatterplots showing correlations of contactin-2 with CSF Aβ42 (A), tTau (B) and pTau (C). The regression lines in the figures are for visualization purposes. r= Spearman’s correlation coefficient. ***p*< 0.01. Since contactin-2 correlated significantly with age within AD group, age correction was performed for correlation analysis in this group. For correlation with Aβ42**:** Corrected r=0.28, *p*=0.02; tTau: Corrected r=0.27, *p*=0.03; *p*Tau: Corrected r=0.32, *p*=0.008.

**Figure S3: Correlations of CSF contactin-2 with CSF neurogranin and BACE1 in validation cohort.** Scatterplot showing correlation of contactin-2 with neurogranin **(A)**. Scatterplot showing correlations of contactin-2 with BACE1 **(B)**. r= Spearman’s correlation coefficient. ***p*< 0.01; ****p*< 0.0001. Since contactin-2 and BACE1 both correlated significantly with age within AD group, age correction was performed. For correlation with neurogranin within AD group: Corrected r=0.30, *p*=0.01. For correlation with BACE1within AD group: Corrected r=0.59, *p*<0.0001.

**Figure S4: Neurogranin and BACE1 levels in Cohort 1 (A-B) and Cohort 2 (C-D).** The values are presented as medians with inter-quartile ranges. **p*< 0.05; ****p*< 0.0001.

**
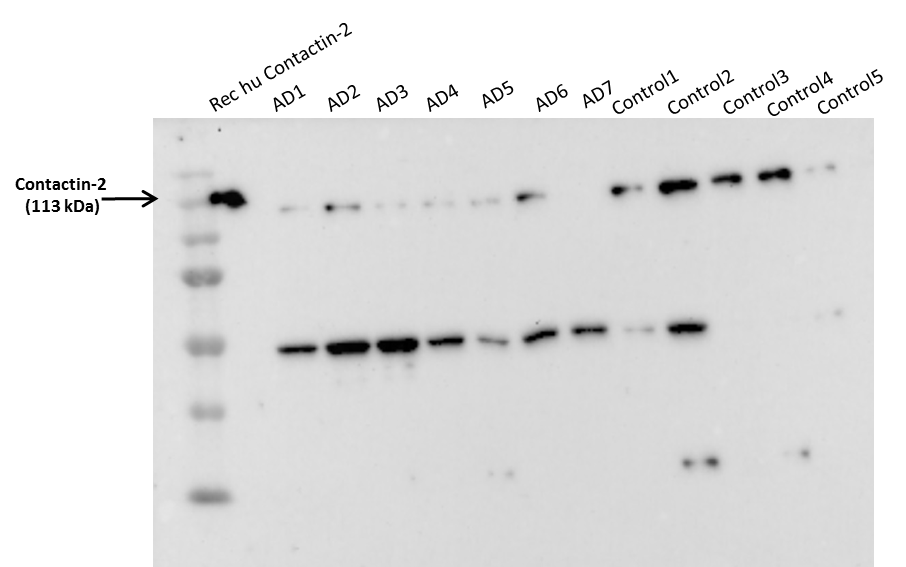
**

**Figure S5: Full Western-blot showing contactin-2 bands.** Lane 2 is human recombinant contactin-2 (positive control). The contactin-2 band appears higher than 113 kDa because it is glycolsylated.


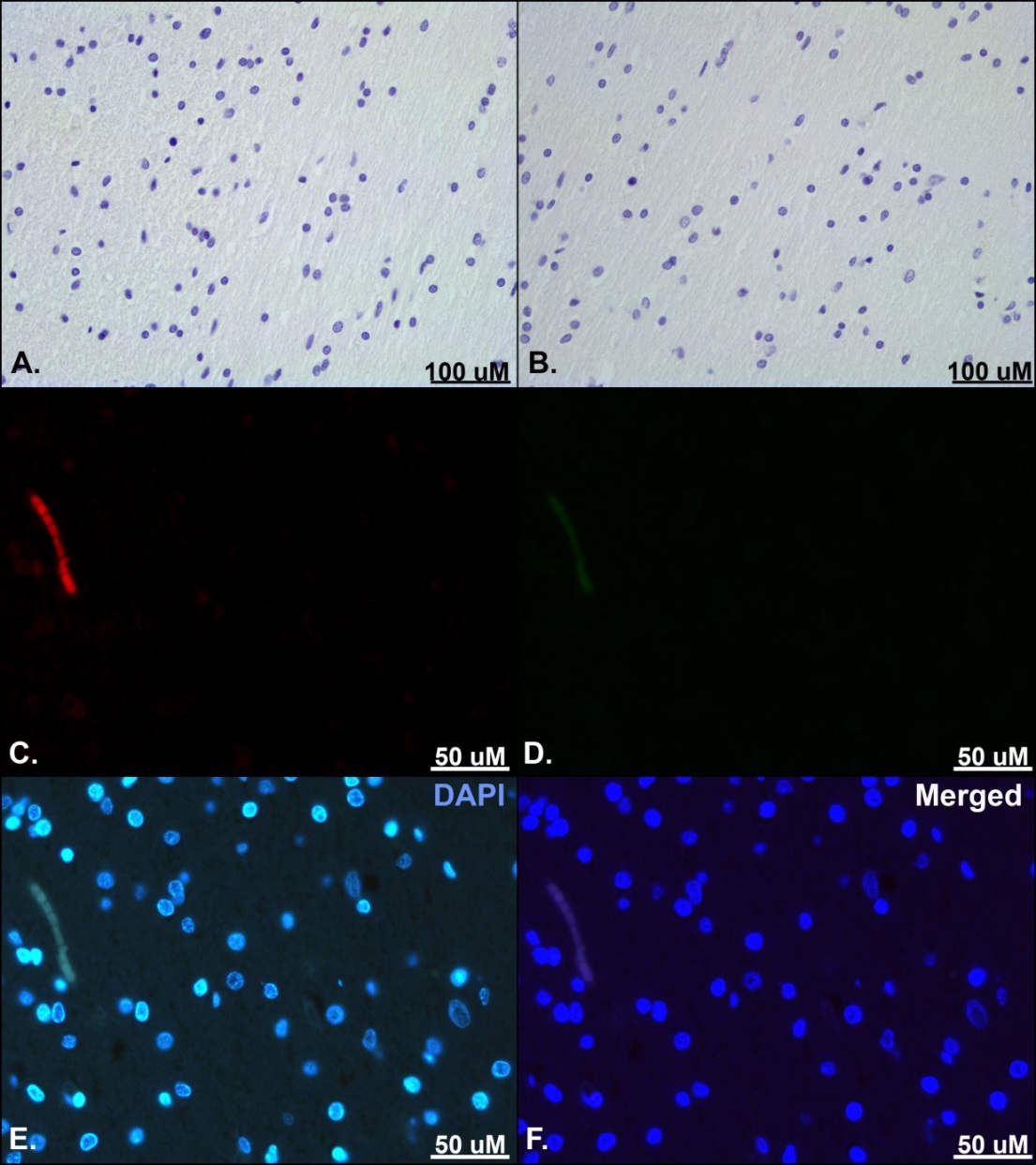


**Figure S6: IF and IHC negative controls.** CA subiculum area of hippocampus (A) and temporal cortex (B) stained with HRP conjugated secondary antibody and H&E counterstain. CA subiculum area of hippocampus stained with red and green fluorophore conjugated secondary antibodies (corresponding to anti-contactin-2 and anti-IC16 primary antibodies) (C, D) and DAPI (E).

**Figure S7: Correlations of CSF contactin-2 levels with tTau in cohort 1.** The regression lines in the figures are for visualization purposes. r=Spearman’s correlation coefficient.

**References**

Braak, H, and E Braak. 1991. “Alzheimer’s Disease Affects Limbic Nuclei of the Thalamus*.” *Acta Neuropathol* 81: 261–68.

Braak, Heiko, Irina Alafuzoff, Thomas Arzberger, Hans Kretzschmar, and Kelly Tredici. 2006. “Staging of Alzheimer Disease-Associated Neurofibrillary Pathology Using Paraffin Sections and Immunocytochemistry.” *Acta Neuropathologica*. doi:10.1007/s00401-006-0127-z.

Reisberg, B, S H Ferris, M J de Leon, and T Crook. 1982. “The Global Deterioration Scale for Assessment of Primary Degenerative Dementia.” *American Journal of Psychiatry* 139 (9): 1136–39. doi:10.1176/ajp.139.9.1136.

Thal, Dietmar R, Udo Rüb, Mario Orantes, and Heiko Braak. 2002. “Phases of A Beta-Deposition in the Human Brain and Its Relevance for the Development of AD.” *Neurology* 58 (12): 1791–1800.
